# Research advances on epigenetic modifications in dendritic cells in allergic rhinitis

**DOI:** 10.3389/fimmu.2025.1682821

**Published:** 2025-10-30

**Authors:** Siyu Duan, Ziyi Jia, Leilei Zheng, Yisha Wu, Zhihan Xu, Haiyi Peng, Jinmei Xue

**Affiliations:** ^1^ Department of Otolaryngology, Head and Neck Surgery, Second Hospital, Shanxi Medical University, Taiyuan, Shanxi, China; ^2^ Shanxi Key Laboratory of Rapid Diagnosis and Precision Treatment of Airway Allergic Diseases, Head & Neck Surgery, Second Hospital, Shanxi Medical University, Taiyuan, Shanxi, China; ^3^ Shanxi Airway Inflammatory Diseases Neuroimmunity Laboratory, Head & Neck Surgery, Second Hospital, Shanxi Medical University, Taiyuan, Shanxi, China; ^4^ Engineering Research Center of AI Intelligent Big Data Platform for Prevention, Treatment and Demonstration Application of Airway Allergy of Shanxi Province, Head & Neck Surgery, Second Hospital, Shanxi Medical University, Taiyuan, Shanxi, China; ^5^ Shanxi Medical University, Taiyuan, Shanxi, China

**Keywords:** allergic rhinitis, dendritic cells, epigenetics, DNA methylation, histone modifications, non-coding RNAs

## Abstract

Allergic rhinitis (AR), a globally prevalent allergic airway disorder, fundamentally involves CD4+T cell subset imbalance, notably T helper 2 (Th2) hyperpolarization. As critical antigen-presenting cells bridging innate and adaptive immunity, dendritic cells (DCs) contribute to the pathogenesis of AR by presenting antigens, modulating T cell differentiation, and regulating inflammatory responses. Emerging research highlights epigenetic alterations within DCs-encompassing DNA methylation, histone modifications, and non-coding RNAs (ncRNAs)-as central molecular mechanisms governing their function and contributing to AR-related immune dysregulation. DNA methylation dynamically regulates promoter regions, influencing DC migration, maturation, and T cell polarization, while also potentially contributing to transgenerational susceptibility to AR, though evidence in humans remains limited. By altering chromatin structure, histone modifications reprogram gene expression networks. This epigenetic remodeling modulates the transcription of inflammation-associated genes within DCs, thereby influencing the balance between immune tolerance and activation. ncRNAs post-transcriptionally regulate DC developmental trajectories, activation thresholds, and signaling pathways, thus impacting Th1/Th2 immune balance. This study aims to systematically review recent advances in research on epigenetic modifications in DCs, provide an in-depth analysis of their mechanistic role in the immune dysregulation of AR, elucidate the molecular basis of their function as both an “environment-gene bridge” and a “transgenerational inheritance vector”, identify current research limitations, and suggest future directions. The goal is to offer a theoretical framework focused on DC epigenetic regulation for understanding AR pathogenesis and developing novel intervention strategies. Unlike previous reviews on epigenetics in allergic diseases, which broadly discuss various immune cells, this study specifically focuses on DCs. This focus is justified because DCs are pivotal initiators and regulators of type 2 immune responses, and their epigenetic status directly dictates the initiation strength and duration of allergic reactions. Furthermore, we clarify the molecular logic of the dual mechanisms mentioned above: DC epigenetics serves both as a molecular translator converting environmental signals into gene regulatory outcomes and as a mechanism for transmitting allergic susceptibility across generations without altering the DNA sequence. Finally, we analyze the barriers to the clinical translation of DC epigenetics-targeted therapies, thereby offering a new perspective from translational medicine for shifting the treatment paradigm of AR from symptomatic control toward immune remodeling.

## 1 Introduction

Affecting over 400 million individuals worldwide, allergic rhinitis (AR) poses significant socioeconomic challenges (1, 2). The key immunological mechanism of AR is an imbalance in the functional subsets of CD4+T cells, dominated by the hyperpolarization of T helper 2 (Th2) cells, leading to immune dysregulation (3). Dendritic cells (DCs) are crucial coordinators between innate and adaptive immunity (4–6). Immature DCs internalize allergens and mature, a transition marked by increased co-stimulatory molecule and MHC levels. Post-migration to lymph nodes, the matured DCs then engage in T cell priming (7). This sequence orchestrates Th2-biased inflammation (1, 8). Unlike other immune cells, DCs stand at the crossroad between allergens and environment, epithelial cells, and immune effector cells (9). They serve as a key initiating link and regulatory hub in the pathogenesis of AR, influencing the progression and outcome of airway allergic diseases (1, 8). It is now clear that DCs in allergic patients exhibit functional abnormalities (9), which may be related to their epigenetic changes. Given the crucial role of DCs in AR and the high plasticity of their functions, the mechanisms of their epigenetic regulation have become an important area for understanding the pathogenesis of AR and identifying intervention targets.

Epigenetics involves heritable, reversible gene function changes independent of DNA sequence alterations, primarily through DNA methylation, histone modifications, and non-coding RNAs (ncRNAs) (10). These modifications dynamically regulate gene expression, essential for establishing and maintaining cellular identity (11). Environmental factors can trigger epigenetic mechanisms that modulate disease susceptibility (12). Epigenetic dysregulation, extensively characterized in tumors, critically influences tumor biology and therapeutic outcomes. By altering gene expression patterns, aberrant epigenetic modifications drive tumor cell proliferation and impair immune cell function. This dynamic remodeling of the tumor immune microenvironment ultimately fuels cancer progression and confers resistance to immunotherapy (13). Advances in epigenetic research have elucidated the pivotal role of these modifications in AR pathogenesis, particularly in shaping individual susceptibility (10). Compared with tumors, although AR has a distinct biological background, a similar principle applies: aberrant epigenetic modifications in DCs can reprogram their functions (such as impaired antigen presentation or skewed T-cell polarization), which shares similarities with the mechanism by which epigenetic disruptions in tumors disturb cellular homeostasis. A growing body of evidence indicates that epigenetic modifications of DCs may represent the core molecular mechanism regulating their functions and are deeply involved in the immune dysregulation of AR (10, 14).

As members of the airway allergic disease spectrum, AR and asthma exhibit shared etiological factors, immunological mechanisms, and pathogenic pathways (15, 16). Their frequent comorbidity results in bidirectional modulation of disease progression (17, 18). So they are considered as “one airway, one disease” (19). In view of this, when elaborating on the role of epigenetic modifications in DCs in AR, this article will appropriately cite important studies in the field of asthma as evidence and complement to more fully understand the relevant mechanisms. This review aims to systematically review the progress of epigenetic modification research in DCs, explore in-depth its mechanism of action in AR immune imbalance, and evaluate the diagnostic or therapeutic predictions that arise from the focus on DC-epigenetics (e.g. candidate biomarkers, drugs, delivery routes…).

## 2 Central role of DCs in the pathogenesis of AR

DCs are critical innate-adaptive immunity interfaces, balancing immune responses and tolerance (4–6). While mainly hematopoietic stem cell-derived in the bone marrow, some DC subsets differentiate from monocytes (20). Myeloid lineage commitment to DCs requires fms-like tyrosine kinase 3 receptor ligand (Flt3L)-dependent signaling (21).DCs are broadly categorized as conventional DCs (cDCs; subdivided into cDC1 and cDC2), plasmacytoid DCs (pDCs), and monocyte-derived DCs (moDCs). cDC1s excel in cross-presentation to activate CD8+ T cells, while cDC2s utilize MHC class II to prime CD4+ T cells with costimulation (6). pDCs initiate host Th1 responses against viral and fungal infections and limit viral replication by secreting type I interferons (IFN-I) (22, 23). moDCs arise from monocytes during inflammation, capable of boosting CD8+ T cell responses or promoting regulatory T cells (Tregs) for immune suppression (6). Following allergen capture, immature DCs mature, upregulating costimulatory molecules and MHC, then migrate to lymph nodes to present antigen to T cells (7). Given their central roles in antigen handling and immune regulation, DC dysfunction directly disrupts immune homeostasis, a well-established feature in AR pathogenesis.

**Figure 1:**
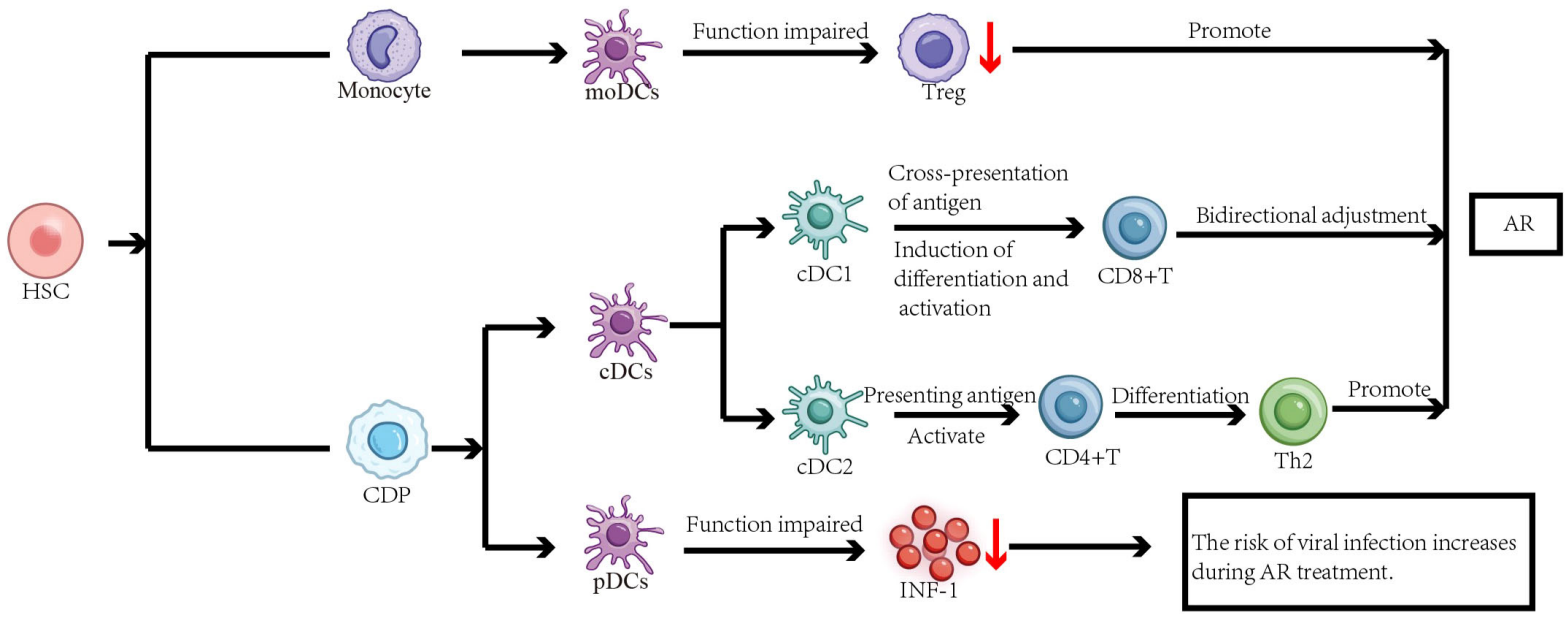
The functions of different dendritic cell subsets and their roles in AR. Both monocytes and common dendritic cell progenitors (CDPs) are derived from hematopoietic stem cells (HSCs). Monocytes can further differentiate into moDCs, while CDPs give rise to cDCs and pDCs.cDC2s present allergens to naive CD4^+^ T cells, leading to their activation and differentiation into Th2 cells, thereby promoting the development of AR.cDC1s play a dual regulatory role in AR pathogenesis by cross-presenting antigens and inducing the differentiation and activation of CD8^+^ T cells. When moDCs function is impaired, the number of activated Treg cells decreases, compromising immune tolerance and amplifying the Th2 response, which exacerbates AR. Dysfunctional pDCs produce less IFN-I, which may be associated with an increased risk of viral infection during AR treatment. Together, these DC subsets contribute to immune dysregulation in AR.

Current research has established that DCs play a significant role in the pathogenesis of AR (1, 8). The aforementioned mechanisms mediated by various DC subsets do not function in isolation but rather constitute a coordinated network that collectively disrupts immune homeostasis and promotes the characteristic Th2-polarized immune imbalance in AR. As the paramount professional antigen-presenting cells, cDC2s play a pivotal role in initiating the adaptive immune response by directly presenting allergens to naive CD4+ T cells via MHC-II, providing co-stimulatory signals, and secreting cytokines (e.g., TSLP, IL-4), thereby directly driving Th2 differentiation. Concurrently, dysfunctional pDCs, characterized by impaired IFN-I production, fail to counteract this Th2 bias and maintain antiviral defense, further tilting the balance. Under inflammatory conditions, recruited moDCs undergo large-scale expansion and enhance the pro-inflammatory milieu, amplifying the Th2 response (**Figure 1**). Furthermore, He et al. found that ovalbumin (OVA) can induce autophagy in bone marrow-derived DCs (BMDCs). This autophagic process enhances the antigen-presenting function of BMDCs, as evidenced by the increased surface expression of class II MHC molecules. Simultaneously, autophagy in BMDCs disrupts the balance of immune cells and related cytokines, promoting a typical Th2 polarization, thus contributing significantly to the development and progression of AR (24, 25). Furthermore, OVA-induced pyroptosis in BMDCs affects CD4+ T cell differentiation and the levels of associated cytokines, leading to an imbalance in Th1/Th2/Th17 cells and promoting AR (26). Additionally, myeloid dendritic cells (mDCs) can promote the production of Th2 cytokines by activating group 2 Innate lymphoid cells (ILC2s), which is related to the occurrence and development of AR (27). Therefore, the pathophysiological processes and dysregulated functions of DCs occupy a crucial position in the immunopathology of AR. Characterizing epigenetic mechanisms as principal molecular determinants provides essential insights into AR immunopathogenesis. It is important to note that key findings, such as OVA-induced autophagy and pyroptosis in BMDCs, are primarily derived from murine experimental models. While these models provide invaluable mechanistic insights, their direct relevance to human AR pathophysiology necessitates further validation. The translation of these mechanisms to human pathology requires confirmation in DCs derived from AR patients’ nasal mucosa or peripheral blood.

## 3 Epigenetic mechanisms

Epigenetic modifications entail reversible changes in gene function without altering the DNA sequence, primarily encompassing DNA methylation, histone modifications, and ncRNAs (10). By modulating chromatin accessibility, these mechanisms control gene expression, influence cellular behavior, shape organismal phenotypes, and critically contribute to disease onset and progression (12, 28).

DNA methylation’s role in gene regulation was firmly established in the 1980s (29, 30). The DNA methylation mechanism involves DNA-methyltransferases (DNMTs)-catalyzed transfer of S-adenosylmethionine-derived methyl groups to cytosines in CpG dinucleotides (31). DNMT1 predominantly methylates hemimethylated replication sites (maintenance methylation) (32), contrasting with DNMT3A/DNMT3B-mediated *de novo* methylation (33). DNMT2’s function remains less defined due to its low activity (34). DNA methylation typically represses transcription by obstructing transcription factor binding and hindering transcriptional machinery access.

Given the repressive effect of DNA methylation on gene expression, DNA demethylation, as its counter-regulatory mechanism, holds equally significant biological importance in maintaining epigenetic homeostasis. Several potential mechanisms of DNA demethylation have been proposed. For example, 5-methylcytosine (5mC) can be deaminated to thymine (T), and demethylation is achieved through DNA glycosylase-mediated G/T mismatch repair. Hydroxylation mediated by ten-eleven translocation methylcytosine dioxygenases (TETs) provides another route for DNA demethylation, producing 5-hydroxymethylcytosine (5hmC) and 5-formylcytosine (5fC) (35–37). These dioxygenases, namely TET1, TET2, and TET3, are critical regulators of DNA demethylation during development and in disease processes including inflammation and oncogenesis (38). Cellular differentiation entails dynamic shifts in DNA methylation, orchestrated by the opposing functions of DNMTs (adding methyl groups) and TET enzymes (initiating demethylation) (39).

Histones, evolutionarily conserved basic proteins, bind nucleic acids to compact the extensive human genome (spanning meters) into densely packed nucleosomes. A nucleosome consists of an octamer of eight histone proteins, around which approximately 145-147 base pairs of DNA are wrapped (40). It is composed of two copies each of four distinct histones (H3, H4, H2A and H2B). Each histone possesses a structured globular core and unstructured N-terminal tails of varying lengths (40). These N-terminal tails undergo various post-translational modifications, including methylation, acetylation, phosphorylation, ubiquitination, SUMOylation, ribosylation and deimination (31).

**Figure 2:**
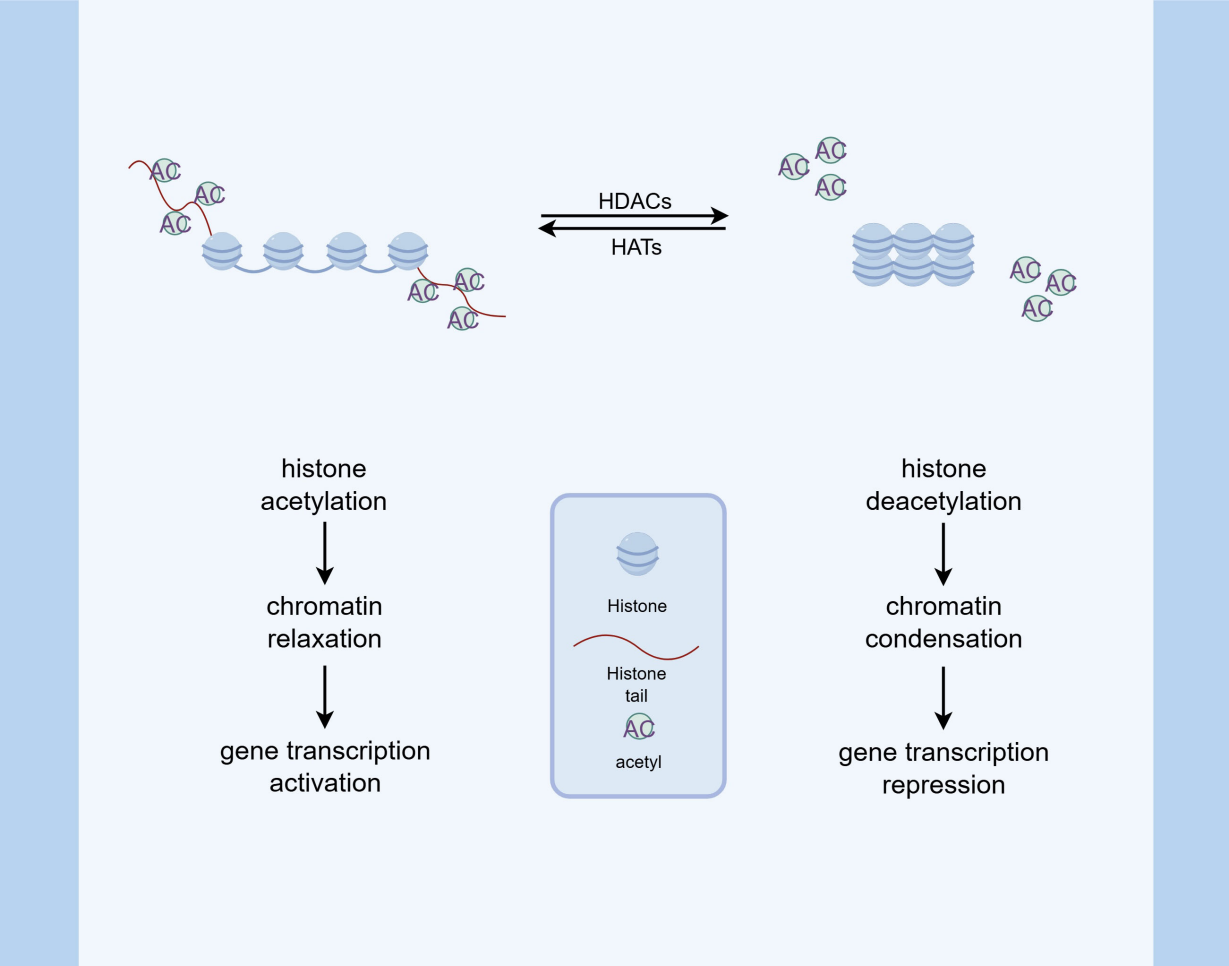
Histone acetylation modification. HATs catalyze the addition of acetyl groups to lysine residues on histone tails, which neutralizes the positive charge of histones and weakens their interaction with negatively charged DNA. This process promotes chromatin relaxation and facilitates the binding of transcriptional activators, thereby enhancing gene expression. Conversely, HDACs remove acetyl groups, leading to chromatin condensation and transcriptional repression. The balance between HAT and HDAC activity is critical in modulating inflammatory responses and other cellular processes.

Histone acetylation, in particular, has been extensively studied and is generally associated with transcriptional activation. In this process, histone acetyltransferases (HATs) covalently attach acetyl groups to lysine residues. This facilitates transcription through two primary mechanisms: neutralizing the positive charge on lysine to weaken non-covalent interactions, and recruiting activator proteins to DNA. These events ultimately lead to increased transcription of associated genes. In contrast, acetyl groups are removed from histones by histone deacetylases (HDACs), resulting in decreased gene expression (41). Alterations in the acetylation-deacetylation equilibrium of core histones have become a pivotal factor in the amplification of inflammatory responses (42) (**Figure 2**).

**Figure 3:**
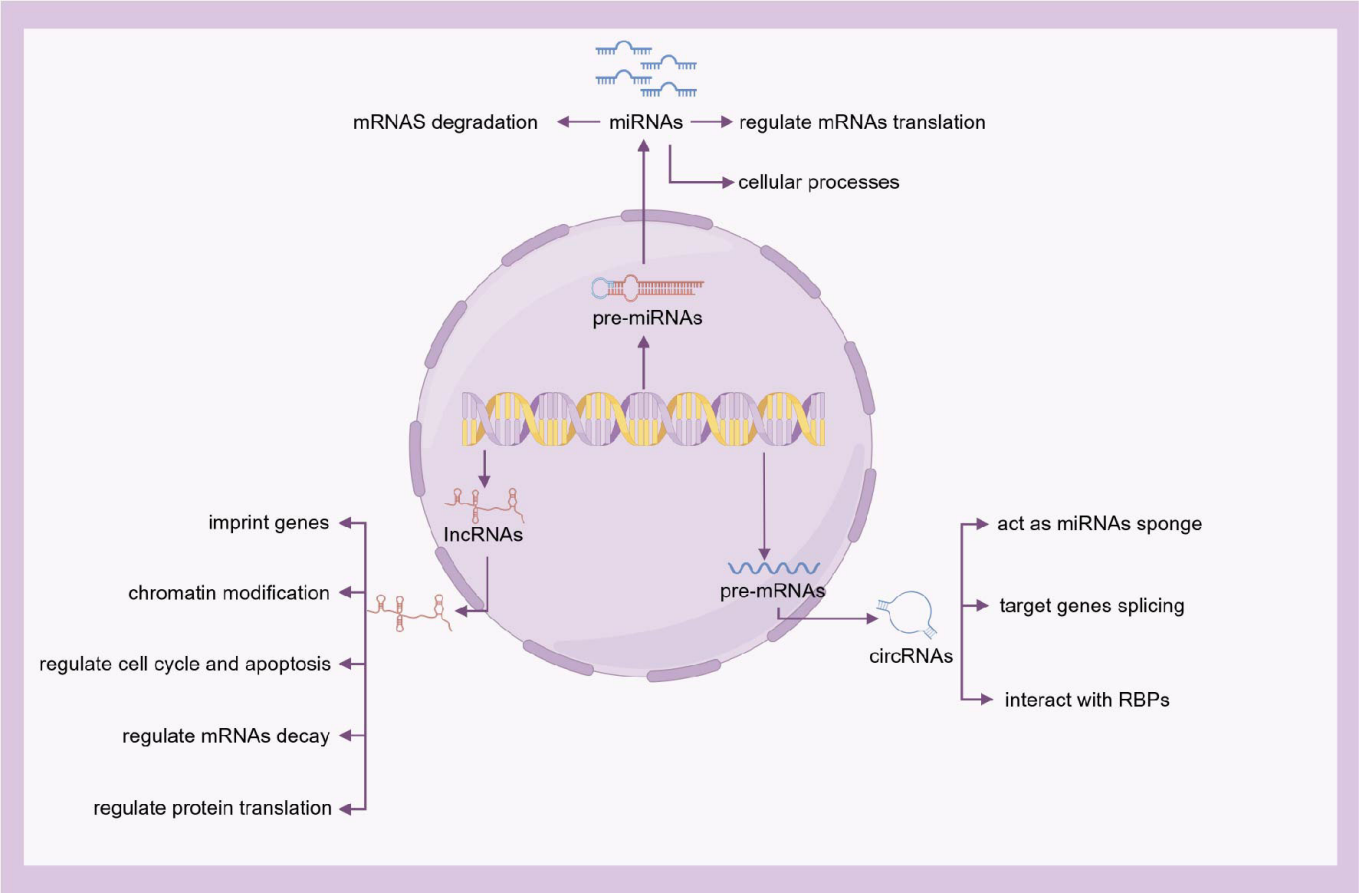
The functions of ncRNAs. On the basis of nucleotide length, ncRNAs are further categorized into lncRNAs, miRNAs, and circRNAs. Although not encoding proteins, ncRNAs serve as crucial regulatory molecules in diverse biological processes.

ncRNAs, although not encoding proteins, serve as crucial regulatory molecules in diverse biological processes. On the basis of nucleotide length, ncRNAs are further categorized into long non-coding RNAs (lncRNAs), microRNAs (miRNAs), and circular RNAs (circRNAs). These ncRNAs play a pivotal role in transcriptional regulation, post-transcriptional regulation, and epigenetic regulation (43) (**Figure 3**).

miRNAs are a class of endogenous ncRNAs that regulate gene expression. These small, highly conserved ncRNAs, approximately 18-24 nucleotides long, suppress target genes at the post-transcriptional level by degrading messenger RNAs (mRNAs) (44). Multiple miRNAs can target the same specific gene, while a single miRNA can target multiple genes within the same cell type, forming complex regulatory networks (45). Numerous recent studies indicate that miRNAs can serve as biomarkers and therapeutic targets for various diseases (46), including allergic diseases (47–49).

circRNAs, typically 100-10,000 nucleotides long, influence gene expression by binding to miRNAs and blocking their regulatory effects on target mRNAs. circRNAs participate in transcriptional activation, post-transcriptional regulation, translation processes, and protein interactions (50).

lncRNAs, defined as transcripts >200 nucleotides, contribute to immune homeostasis regulation and participate in allergic pathologies such as asthma and AR. These molecules represent potential therapeutic targets and diagnostic biomarkers for allergic conditions. These RNAs can induce alterations in chromatin structure and are therefore considered essential epigenetic regulators (51–55).

Subsequent sections examine the functional impact of these epigenetic mechanisms on DCs and their consequent modulation of AR immune responses.

## 4 DNA methylation regulates DC function and AR

DNA methylation-a core epigenetic regulatory process-exerts profound effects on transcriptional activity through dynamic cytosine nucleotide alterations. DC-intrinsic methylation patterning orchestrates key cellular functions including development, maturation, migration dynamics, antigen presentation processes, and immunomodulation (56). This analysis delineates the regulatory significance of DNA methylation/demethylation dynamics in DC functional specification. We will explore how these processes regulate the expression of specific genes to drive Th2 immune polarization and disrupt immune tolerance. Furthermore, we will elucidate their central mechanisms in mediating susceptibility to environmental exposure-induced AR and its transgenerational inheritance risk.

Asthmatic dendritic cells exhibit hypermethylation in the Runx3 promoter region, as evidenced by recent studies. Runx3—a pivotal Runx family transcription factor—governs cell fate determination and modulates diverse biological pathways. Its promoter hypermethylation suppresses expression, impairing DC immunoregulatory capacity and destabilizing immune homeostasis. In Runx3-null murine models, BMDCs show disrupted transcriptional control of C-C chemokine receptor 7 (CCR7). This suppresses DC migration towards CCR7 ligands and results in the development of asthma-like symptoms in mice (57).

Beyond the DNA methylation of specific genes, as heritable epigenetic marks, DNA methylation plays a larger role in the transgenerational transmission of immune risk induced by allergen exposure. In a murine study investigating the impact of maternal allergen exposure on offspring, genome-wide DNA methylation analysis revealed that the splenic CD11c+DCs of offspring from female mice sensitized with OVA in an allergic asthma model exhibited a distinct DNA methylation profile compared to offspring from non-sensitized dams. Specifically, 40 differentially methylated CpG sites were identified, showing an approximately 9-fold or greater difference in DNA methylation between the two offspring groups. Overall DNA methylation levels were higher in the DCs of offspring from sensitized mothers (58, 59). This altered DNA methylation pattern improved the efficiency of DC in allergen presentation, promoting a Th2-skewed immune response, triggering atopic reactions, and increasing susceptibility to asthma (41, 59, 60).

Another study found that maternal exposure to environmental particles such as diesel exhaust particles (DEPs) during pregnancy in mice induced significant DNA methylation alterations in DCs in the F1, F2 and F3 generations. This included 14,480 altered CpG sites in F1, 9,413 in F2 and 6,239 in F3, concomitant with an increased susceptibility to asthma in the offspring. Treatment of F1 female offspring with the DNMT inhibitor decitabine attenuated the asthmatic risk phenotype in subsequent generations, demonstrating a causal role for altered DNA methylation in DCs in the transgenerational transmission of asthma risk (61). Collectively, these findings indicate that epigenetically modified DCs participate in the process by which allergic risk is transferred from the mother to the offspring. These findings derive from murine models, their relevance to human AR remains to be confirmed.

As a counter-regulatory mechanism for DNA methylation, DNA demethylation in DCs also plays a significant role in AR. During *in vitro* differentiation of human monocytes into immature DCs and subsequent maturation of these immature DCs, site and time-specific DNA demethylation occurs. This demethylation is predominantly located near promoters, enhancers, and transcription factor binding sites and is associated with increased expression of nearby genes (39). However, research by Li et al. indicates that DNA is in a hypermethylated state during DC maturation. This hypermethylation results not only from increased expression of DNMT3B, but also from suppression of the DNA demethylation enzyme TET1. Following 48 hours of allergen stimulation, atopic moDCs exhibited significantly reduced TET1 expression alongside an upregulation of costimulatory molecules CD86, CD80, and CD40. Consequently, this impaired the conversion of naive T cells into activated Tregs, thereby compromising the ability of moDCs to maintain immune tolerance and homeostasis in AR (38).Burleson et al. (62) further demonstrated that TET1 deficient mice sensitized with house dust mite (HDM) exhibited impaired interferon pathways, increased airway hyperresponsiveness, increased pulmonary eosinophil infiltration, and exacerbated inflammation. Thus, the loss of TET1 promotes DC maturation and mediates the development of allergic airway inflammation.

In addition, environmental risk factors can alter the epigenome of the DCs and influence the susceptibility of the disease. Parental exposure of C57BL/6 mice to low-dose di-(2-ethylhexyl) phthalate (DEHP) resulted in hypomethylation of the insulin-like growth factor 2 receptor (Igf2r) promoter in DCs of the offspring, leading to increased expression of *IGF2R*. Concurrently, ligand-stimulated CD8α+ DCs exhibited significantly increased apoptosis and reduced IL-12 secretion. This was followed by a decrease in T cell-derived IFN-γ, ultimately promoting Th2-associated allergic responses in the lungs. In the human placenta, DNA methylation levels in the homologous *IGF2R* promoter region were negatively correlated with maternal DEHP intake. This study highlights the importance of parental DEHP exposure in conferring transgenerational risk of an allergic phenotype, characterized by hypomethylation of *IGF2R* and dysregulation of DC homeostasis (63). This represents limited human observational data, and causal links to transgenerational AR have not been established. These findings collectively encourage the exploration of potential environmental factors that mediate the epigenetic regulation of AR. Targeting the modulation of the dynamic balance of DNA methylation in DCs holds promise for developing more effective strategies for the prevention and treatment of AR.

In this section, research findings such as hypermethylation of the Runx3 gene promoter region in DCs, transgenerational inheritance of DNA methylation induced by allergen and environmental particle exposure, and hypomethylation of the IGF2R promoter region promoting airway allergy are derived from asthma models. Given that AR and asthma share the core mechanism of immune imbalance, this DNA methylation status and its impact on DC function are likely to play a similar role in AR, but its applicability remains to be studied.

## 5 Histone modifications in DC-mediated AR

Histone post-translational modifications precisely modulate gene expression by dynamically restructuring chromatin and engaging transcriptional regulators. As pivotal epigenetic determinants, these modifications critically shape dendritic cell-initiated inflammatory networks (64). This section will systematically elaborate how histone modification and its regulatory enzymes in DC affect the expression of key molecules, thus participating in the pathogenesis of AR/asthma and affecting the response to treatment.

Asthma pathogenesis engages mitogen-activated protein kinase (MAPK) inflammatory pathways (65), counterregulated by dual-specificity phosphatase-1 (DUSP1). Epigenetic studies disclose concomitant histone H4 deacetylation and DUSP1 downregulation in asthmatic children’s peripheral DCs. This dysregulation abrogates extracellular signal-regulated kinase 1/2(ERK1/2), c-Jun N-terminal kinase 1(JNK1), and p38 inhibition, intensifying inflammatory pathology (66). It should be noted that this study only reported a correlation between histone H4 deacetylation and downregulation of DUSP1, and has not yet validated a causal relationship through ChIP-seq or functional experiments.

IL-10 has a wide range of anti-inflammatory effects, which can inhibit the production of inflammatory cytokines and reduce inflammation and hyperresponsiveness of the airways. Lipopolysaccharide (LPS) has been found to induce histone H3 acetylation in the promoter region of IL-10 gene in mDCs, and montelukast, an asthma therapeutic drug, can further enhance this process. The suppression of IL-10 expression by treatment with the HDAC inhibitor anacardic acid suggests that montelukast enhances IL-10 expression by promoting histone H3 acetylation, thereby epigenetically regulating the function of mDCs, reducing the expression of maturation markers, and inhibiting the ability of mDCs to stimulate T cells to Th2 type immune responses. This, in turn, reduces airway inflammation (67). Although this mechanism has been preliminarily verified in *in vitro* experiments, it still needs to be further confirmed through ChIP in DCs derived from AR patients.

HDAC dysregulation in DCs governs histone acetylation dynamics, modulating asthma development and treatment efficacy in murine models. Significantly, c-Myc interacting zinc finger protein-1(Miz1) expression is enhanced in pulmonary DCs of asthmatic mice. Further studies showed that Miz1 recruited HDAC1 to the IL-12 promoter region in DCs. This results in histone deacetylation of its promoter and inhibits IL-12 transcription in DCs, thereby promoting Th2 responses in asthma (68). This study confirmed through ChIP experiments that HDAC1 directly binds to the IL-12 promoter, indicating a causal relationship. Oxidative stress caused by environmental factors such as tobacco smoke exposure inactivates HDAC2 through nitrification or carboxylation, phosphorylates, ubiquitinates, and degrades HDAC2, resulting in epigenetic reprogramming of DCs and Th2-biased immune response to inhaled antigens, resulting in airway allergy. Similarly, *in vitro* pharmacological inhibition of HDAC in DC was found to alter Th1/Th2 balance and promote airway allergy by enhancing expression of indoleamine 2,3-dioxygenase in DCs, inhibiting IL-12p40 production, and disrupting DCs-controlled activation of Th1 effector cells (69). This conclusion is mainly based on pharmacological inhibition experiments and gene knockout techniques, but the modification status and function of HDAC2 have not yet been verified in patients with AR. In a murine model of mixed granulocytic asthma, reduced HDAC2 expression on CD11c+DCs in the lung correlates with glucocorticoid resistance during treatment, and pharmacologic restoration of HDAC2 expression on CD11c+DCs reestablishes glucocorticoid sensitivity in a mouse model of asthma (70). This finding suggests that the expression level of HDAC2 may be related to the response to AR treatment, but this has not yet been verified in clinical samples of AR. In addition, Zhang et al. demonstrated that membrane-associated RING-CH1(March1) promotes ubiquitin-mediated degradation of HDAC11 in DCs from pediatric and animal asthma models. This abolished HDAC11’s inhibitory regulation of OX40 ligand (OX40L), consequently enhancing OX40L expression and contributing to asthmatic pathogenesis (71). This study confirmed the interaction between March1 and HDAC11 through Co-IP and ubiquitination experiments, providing strong mechanistic evidence.

In addition to the role of histone acetylation modification in allergic airway diseases, histone methylation in DCs also plays an important role in the treatment and related complications of AR and asthma. It has been found that formoterol inhibits the translocation of methyltransferase WD repeat domain 5 (WDR5) from the cytoplasm to the nucleus in human pDCs, thereby inhibiting the trimethylation of histone H3K4 in the promoter region of IFNA and IFNB genes and reducing the expression of IFN-I. This is associated with an increased risk of infection during the treatment of asthma with formoterol (72). This mechanism still lacks direct evidence in the context of AR. Prostacyclin I_2_ (PGI_2_) analogues are potential drug candidates for the treatment of asthma. Iloprost, an analogue of PGI_2_, modulates cytokine expression in human mDCs via epigenetic mechanisms. Specifically, it impedes the nuclear translocation of the H3K4-specific methyltransferase mixed lineage leukemia (MLL) and WDR5. This blockade results in reduced trimethylation of histone H3K4 within the TNFA gene promoter region, ultimately leading to the suppression of TNF-α production. This revealed that PGI_2_ analogues act on mDCs to exert anti-inflammatory effects through an epigenetic mechanism, providing a new potential target for asthma treatment (73). This study reveals a new mechanism by which drugs exert anti-inflammatory effects through histone methylation modification, providing potential targets for AR treatment. However, it has not yet been verified in the AR model. Enhancer of zeste homolog 2 (EZH2), the catalytic subunit of polycomb repressive complex 2, functions as a histone lysine methyltransferase specific for trimethylating lysine 27 on histone H3 (H3K27me3). This epigenetic modulator plays critical roles in diverse immune cell types, such as B cells, T cells, and DCs (74). Research by Gunawan et al. demonstrated that EZH2 controls DC adhesion and motility via post-translational methylation of Talin1, which subsequently impairs Talin1’s interaction with F-actin (75). Furthermore, Li et al. reported that in patients with AR undergoing allergen immunotherapy (AIT), pharmacological inhibition of EZH2 suppressed DC activation. This suppression was characterized by reduced expression of DC costimulatory molecules and diminished capacity of DCs to stimulate T cell proliferation. Consequently, EZH2 inhibitors represent promising adjunctive agents to enhance AIT efficacy and potentially reduce treatment duration in AR patients (74). This study established a causal role of EZH2 in regulating DC function through *in vitro* inhibition experiments, providing a novel therapeutic strategy for AR.

In histone modifications, the downregulation of DUSP1 in peripheral blood DCs of asthmatic children is associated with histone H4 deacetylation; LPS promotes histone H3 acetylation in the promoter region of the IL-10 in mDCs, thereby enhancing IL-10 expression; and abnormal expression levels or functional impairments of the HDAC family (HDAC1, HDAC2, HDAC11) contribute to the pathogenesis of asthma, as well as the mechanisms of action of drugs targeting the translocation of histone methyltransferases (MLL, WDR5). All these findings are derived from studies on asthmatic children and relevant animal experiments. Given that asthma and AR share commonalities in etiological factors, immune mechanisms, and pathogenic pathways, these histone modification-related mechanisms provide important reference directions for exploring the pathogenesis of AR. However, AR and asthma differ in terms of lesion locations, primary allergen exposure patterns, and local immune microenvironments, so the research conclusions derived from asthma cannot be directly and simply extrapolated to AR.

Specifically, the applicability of the histone modification characteristics of DCs observed in asthma to AR remains to be verified: ① It is necessary to detect the expression level of DUSP1 and the acetylation status of histone H4 in nasal mucosal DCs from AR patients, so as to clarify their association with the severity of AR symptoms; ② It should be explored whether LPS or AR-related allergens (such as dust mites and pollen) affect the immunoregulatory function of DCs in AR patients by regulating histone acetylation in the IL-10 promoter region; ③ It is required to compare the differences in the expression and activity of HDAC family members in DCs between AR and asthma patients, and analyze the efficacy of HDAC-targeted drugs in AR models. Only through such targeted studies can the actual role of these histone modification mechanisms in AR and their clinical transformation value be clarified.

## 6 ncRNAs regulate DCs function and AR immune response

ncRNAs represent a rapidly evolving domain within the epigenetic regulatory network, exerting significant control over gene expression both transcriptionally and post-transcriptionally. Specifically, miRNAs and lncRNAs have been demonstrated to critically influence DC development, activation status, and functional properties (76, 77). Consequently, these ncRNAs profoundly impact the equilibrium among Th1, Th2, and Treg subsets. This section will focus on the expression patterns of several key ncRNAs in DCs and their regulatory networks, clarifying how they participate in the immune imbalance of AR by interfering with DCs function.

miRNAs, a class of short non-coding RNA molecules, function in post-transcriptional gene silencing primarily through inducing the decay of complementary target mRNAs. Some evidence suggests that miRNAs are dysregulated in allergic and asthma diseases (78–80). Accumulating evidence indicates that miRNAs potentially regulate innate immunity. Furthermore, their involvement extends to the establishment of innate immune memory within monocytes and DCs (80, 81).

miRNAs have multiple functions in DCs, and miR-155, Let-7i, and miR-126 are associated with DCs maturation and function. On the contrary, miR-148, miR-142, miR-146a and miR-29a delayed DCs maturation. Specific miRNAs modulate DC functions: miR-150 and miR-223 influence DC-mediated antigen presentation to T cells, while miR-155, miR-146a, miR-126, miR-29b, and miR-29c impact DC survival (76).

miRNAs modulate inflammatory processes through regulating DC development, differentiation, and activation (82). Among immune-related miRNAs, miR-155 has been extensively characterized and linked to allergic conditions. This miRNA is encoded on chromosome 21 (21q21), a locus associated with susceptibility to asthma, pollen allergy, and atopic dermatitis. Studies using *in vitro* knockdown of miR-155 have confirmed that it promotes DC maturation and migration and enhances Th2 responses by targeting c-Fos for suppression. In genetic knockout mouse models, deficiency of miR-155 significantly alleviated allergic airway inflammation (83, 84). Notably, Zech et al. (83) further revealed that miR-155 modulates ATP degradation by targeting ectonucleoside triphosphate diphosphohydrolases (ENTPD1 and ENTPD3), thereby influencing the purinergic receptor (P2R) signaling pathway in DCs. Their study, conducted in miR-155-deficient (miR-155^-^/^-^) mice using both OVA and HDM-induced allergic airway inflammation (AAI) models, demonstrated that miR-155 deficiency impaired ATP-directed chemotaxis of DCs and reduced IL-1β secretion. These effects were accompanied by increased expression of ENTPD1 and ENTPD3 and enhanced ATPase activity, thereby mechanistically explaining how miR-155 influences Th2 polarization and airway inflammation via regulation of the ATP-P2R signaling axis in DCs. Separately, Luo et al. demonstrated that IL-4 upregulates miR-19a expression in DCs, which in turn suppresses IL-10 production—a mechanism implicated in the pathogenesis of AR and nasal polyposis. Notably, this finding was substantiated using DCs derived from patients with AR, where miR-19a levels were significantly elevated and negatively correlated with IL-10 expression. Further *in vitro* functional validation showed that knockdown of miR-19a via RNA interference abolished IL-4–induced suppression of IL-10, supporting a causal role of this miRNA in disrupting immune tolerance (85).

miRNAs not only can promote allergic response, but some of them can also affect DCs in helping to establish immune tolerance or inhibiting allergic reactions. Studies have demonstrated that miR-106b participates in the regulation of DC development by directly binding to the 3′-untranslated region (3′-UTR) of early growth response factor 2 (Egr-2) and suppressing its expression (86). This mechanism has been validated by luciferase reporter assays, in which miR-106b mimics significantly inhibited luciferase activity in HEK293 cells carrying the wild-type Egr-2 3′-UTR, but not a mutated version. Functionally, miR-106b modulates the maturation status and surface marker expression (e.g., MHC II, CD80, CD86) of DCs, thereby influencing their antigen-presenting capacity and immune-activating functions. Of particular note, miR-106b expression was significantly downregulated in OVA-activated BMDCs. Transfection with a miR-106b inhibitor further promoted BMDC maturation and surface co-stimulatory molecule expression, while significantly suppressing IL-12 secretion. Conversely, overexpression of miR-106b mimics enhanced IL-12 production and promoted Th1 polarization. These findings suggest that miR-106b contributes to the regulation of DC-mediated Th1/Th2 immune balance by targeting Egr-2 and modulating IL-12 secretion, thereby playing an important role in allergic immune dysregulation. However, it should be noted that this mechanism is primarily based on *in vitro* murine BMDC models and lacks direct functional validation in DCs derived from AR patients (86). *In vitro* experiments using bone marrow-derived DCs co-cultured with naive T cells, coupled with *in vivo* studies in an AR mouse model, demonstrated that miR-146a promotes TGF-β production in DCs, which subsequently drives the differentiation of naive CD4^+^ T cells into Tregs and ultimately potentiates the efficacy of OVA-specific immunotherapy (87). miR-223 plays a role in suppressing DCs activation during inflammation and maintains an antimature state, thereby promoting immune tolerance responses (88). Research demonstrates that miR-23b augments the tolerogenic function of OVA-exposed DCs through dual suppression of the Notch1 and nuclear factor κB (NF-κB) signaling pathways (84).

Another important ncRNAs are lncRNAs, which are more than 200 nucleotides in length and do not function as protein coding. This RNA is seen as an indispensable epigenetic regulator that may be involved in the biological behavior of cells (89). Analysis revealed 962 differentially expressed lncRNAs in DCs between AR patients and healthy controls, comprising 434 upregulated and 528 downregulated transcripts. Altered expression of these lncRNAs likely modulates DC differentiation, maturation, and antigen-presenting capacity, consequently influencing the immune response underlying AR. Through transcriptomic analysis of DCs derived from patients with AR, it was found that differentially expressed mRNAs were significantly enriched in several key immune pathways. Among them, pathways such as IFN-γ signaling, membrane repolarization, and peptide antigen binding are closely associated with the physiological state of DCs. The study further suggested, via co-expression network analysis, that dysregulation of these pathways may be related to the modulation by specific lncRNAs, which could influence the expression of key genes within these pathways through cis- or trans-acting mechanisms. Thus, lncRNAs may regulate phagocytic and antigen-presenting functions of DCs through the aforementioned pathways, thereby contributing to immune dysregulation in AR (89). However, these computationally predicted associations still require further experimental validation. FOXD3-AS1, as a lncRNA, can inhibit the maturation of DCs and increase the Th1/Th2 cell ratio by inhibiting STAT6 phosphorylation, thereby improving AR symptoms (90). Nevertheless, the precise biological functions and molecular mechanisms underlying the vast majority of dysregulated lncRNAs in AR remain largely unexplored. Elucidating these aspects constitutes a critical future research direction, providing a foundation for developing lncRNA-based diagnostic biomarkers and targeted therapeutics.

Although numerous ncRNAs have been identified as differentially expressed in AR, their causal roles remain largely unverified, with most currently serving as correlative biomarkers. Future studies should prioritize the use of genetic and pharmacological tools in AR-relevant models to perform causal validation, thereby distinguishing bona fide pathological drivers from secondary effects.

## 7 Conclusions and prospects

This review synthesizes current knowledge on the central role of epigenetic regulation—encompassing DNA methylation, histone modifications, and ncRNAs—in DCs and its contribution to immune dysregulation underlying AR. The studies discussed herein highlight how environmentally-driven epigenetic reprogramming of DCs modulates their maturation, migration, cytokine production, and T-cell polarizing capacity, thereby critically influencing AR pathogenesis and therapeutic responses.

Among these mechanisms, DNA methylation currently possesses the strongest evidence base in the context of AR, particularly due to its well-documented role in transgenerational inheritance and response to environmental exposures. Studies in murine models have consistently demonstrated that allergen or particulate matter exposure leads to heritable changes in DNA methylation patterns within DCs, which correlate with enhanced Th2 responses and increased asthma susceptibility (58–61). In contrast, evidence for histone modifications and ncRNAs in AR remains more preliminary, largely extrapolated from asthma models or *in vitro* studies, and requires further validation in human AR-specific contexts.

However, a critical appraisal of the available evidence necessitates an important caveat: a substantial portion of the mechanistic data—particularly regarding Runx3 promoter hypermethylation, HDAC dysregulation, and the functions of specific ncRNAs—originates from studies conducted in experimental asthma models. Although asthma and AR share common etiological factors and immune pathways (e.g., Th2 polarization, DC activation, and epithelial barrier dysfunction), they are distinct clinical entities with tissue-specific microenvironments and inflammatory profiles. The nasal mucosa in AR exhibits unique anatomical and immunological features that may not fully mirror the bronchial environment in asthma. Therefore, while extrapolation from asthma studies provides invaluable hypotheses and mechanistic insights, direct evidence from AR-specific models and human studies is imperative to confirm these epigenetic mechanisms in the context of rhinitis. Future research must prioritize the validation of these findings in AR-relevant systems—such as nasal epithelial cells, local DC subsets, and patient-derived samples—to establish a more definitive and disease-specific epigenetic landscape. This will not only enhance the translational relevance of epigenetic therapies but also solidify the evidence base for AR.

DCs subsets play distinct functions in AR, and their epigenetic regulatory mechanisms differ: In cDCs, DNA methylation silences Runx3 expression, impairing their immune functions and disrupting the balance between immune response and tolerance (57). pDCs downregulate IFN-I through aberrant histone methylation, such as WDR5-mediated inhibition of H3K4 trimethylation (72), which compromises their antiviral capacity and elevates the risk of infections during asthma treatment. In moDCs, defective DNA demethylation caused by reduced TET1 expression inhibits their ability to promote the differentiation of naive T cells into activated Tregs, resulting in a loss of immune tolerance (38). This subset-specific epigenetic heterogeneity underscores the urgency for developing precision therapeutic strategies. Current epigenetic research predominantly focuses on mixed DC populations or individual subsets, and lacks systematic comparison of the epigenetic states across different DC subsets in AR patients. Future studies should employ single-cell multi-omics technologies to directly analyze the epigenetic features in various DC subsets derived from the nasal mucosa and peripheral blood of AR patients. Furthermore, such heterogeneity suggests that epigenetic interventions must be subset-specific rather than employing a pan-DC strategy: for example, targeting Runx3 methylation in cDCs may require demethylating agents, whereas correcting aberrant histone modifications in pDCs would necessitate inhibitors targeting specific histone methyltransferases.

Although the epigenetic regulation of DCs provides new targets for AR treatment (such as DNMT inhibitors and EZH2 inhibitors), its clinical application faces challenges (**Table 1**). First, the tissue-specific barrier, that is, it is difficult to accurately target nasal mucosal DCs by systemic drug delivery, and the technology of nanoparticle delivery and local drug delivery needs to be broken through urgently. Second, there is a bottleneck in biomarker mining, as existing studies focus on animal models and population data are scarce. Combined with single-cell multi-omics, the apparent characteristic profiles of human DCs can be screened and their value as predictive markers for efficacy can be verified. However, the implementation of this approach requires careful consideration of practical feasibility and ethical challenges. (i) Sample acquisition represents a critical bottleneck: the collection of nasal mucosal DCs involves invasive procedures with high technical difficulty, which limits patient recruitment. Although peripheral blood samples are more accessible, they may not fully capture the epigenetic features of tissue-resident DCs within the local immune microenvironment. (ii) Single-cell sequencing places extremely high demands on sample quality, library preparation protocols, and data analysis capabilities, accompanied by substantial costs. (iii) Rigorous ethical review and fully informed consent are essential, explicitly covering genomic analyses and potential future research uses. Addressing these challenges will require multidisciplinary collaboration to establish standardized protocols and ensure ethical compliance. Thirdly, epigenetic drugs may have off-target effects and long-term safety issues. Finally, there are individual differences in epigenetic profiles, and how to achieve precise intervention is still a topic that needs to be studied.

**Table 1:** Potential epigenetic targets, corresponding drug classes, supporting evidence, and limitations in DCs for AR.

Potential targets	Drug classes	Supporting evidence	Limitations
DNMTs	DNMT inhibitors (e.g.,decitabine)	After maternal mice were exposed to DEPs during pregnancy, the offspring showed abnormal DNA methylation in DCs. Treatment of F1 female offspring with DNMT inhibitors could attenuate the asthmatic risk in subsequent generations (61).	Evidence mostly from animal models; limited human data; systemic administration may disrupt normal cellular DNA methylation homeostasis.
TETs	TET activators (under development)	Reduced TET1 expression in DCs inhibited Treg differentiation and promoted AR inflammation (38); TET1-deficient mice exhibited exacerbated allergic airway inflammation (62).	No specific TET activators available; role in human AR unvalidated.
HDACs	HDAC inhibitors	HDAC1 induces histone deacetylation at the IL-12 promoter region, which inhibits IL-12 transcription in DCs and promotes the Th2 response in asthma (68).	Poor tissue specificity; may disrupt normal immune regulation; potential off-target effects with long-term use.
EZH2	EZH2 inhibitors	EZH2 inhibitors suppressed DC activation, reduced co-stimulatory molecule expression, and enhanced AIT efficacy in AR (74).	Only preliminarily validated in AIT; efficacy as monotherapy in AR unknown.
miR-155	Anti-miR-155 oligonucleotides	miR-155 promoted DC maturation and Th2 differentiation via c-Fos silencing; miR-155 ablation alleviated allergic airway responses (83, 84).	Oligonucleotide delivery systems lack target specificity; may interfere with miR-155 function in other tissues.

In conclusion, the epigenetic regulatory network in DCs is an important hub connecting genetic susceptibility, environmental exposure and AR immunophenotype. Future research should focus on using high-throughput single-cell epigenomics to map the whole genome of DCs subsets in AR patients and explore the synergistic effect of epigenetic-related target drugs with existing AR therapies. The transformation of AR from symptom control to immune reprogramming can only be realized by in-depth understanding of the dynamic hierarchy of the epigenetic network in DCs.
